# Endogenous attention is invariant to sequential effects in performance around the visual field

**DOI:** 10.64898/2026.07.18.739376

**Published:** 2026-07-22

**Authors:** Hsing-Hao Lee, Marisa Carrasco

**Affiliations:** 1 Department of Psychology, New York University, New York, NY 10003, USA; 2 Center for Neural Science, New York University, New York, NY 10003, USA

**Keywords:** contrast sensitivity, covert spatial attention, endogenous attention, sequential effect, visual field asymmetries

## Abstract

Covert spatial attention selects and prioritizes relevant sensory information. Endogenous attention is voluntary, goal-driven, and flexible. However, it cannot alleviate visual polar-angle asymmetries, specifically, the horizontal-vertical anisotropy and the vertical meridian asymmetry. Visual perception is affected by both current sensory inputs and contextual information over time and space, such as the perception of preceding trials. Previous studies reported sequential effects whereby attention interacts with response repetitions. But it is unknown whether and how endogenous attention modulation on performance varies as a function of target location and trial history. Here, we reanalyzed data from three published studies of endogenous attention in orientation discrimination tasks, to (1) assess the typical sequential effects on response, in which response to the current trial is biased toward the previous one, and (2) examine if sequential effects would modulate the performance across locations, across four dimensions: (1) location, (2) feature, (3) attention repetition condition, and (4) the correctness of the preceding (*n* −1) trial. First, we demonstrated typical sequential effects of response repetition to the repeated location and feature aspects of the target. Second, we found a robust effect of attention on performance, but the results did not reveal evidence of sequential attention effects as a function of the four dimensions in any of the three studies. Moreover, there were no interactions between attention and location when considering trial history. Together, these findings provide compelling evidence that visual polar-angle asymmetries are resistant to endogenous attention, and that even top-down factors–sequential effects–do not alleviate these asymmetries in performance.

## Introduction

Visual perception depends on sensory input and is modulated by its location across the visual field, as well as by sequential history and covert attention. In this study, we investigated whether and how sequential effects influence endogenous attentional modulation across the visual field, particularly around polar angle.

Visual perception depends not only on current sensory input but also on contextual information over space and time (Cicchini et al., 2024; Fecteau & Munoz, 2003; Fischer & Whitney, 2014; Kiyonaga et al., 2017; Manassi et al., 2023; Manassi & Whitney, 2024; Pascucci et al., 2023). To combat environmental noise and stabilize visual input, response and perception at the present moment are biased toward input from the recent past. For example, the perception of an oriented grating in the current trial is biased toward the orientation of the grating in the previous trial. This phenomenon—serial dependence or the sequential effect—has been shown in orientation (Bliss et al., 2023; Can & Collins, 2025; Collins, 2019; Fischer & Whitney, 2014; Fritsche et al., 2017; Gekas et al., 2019; Little & Clifford, 2025; Murai & Whitney, 2021; St. John-Saaltink et al., 2016), color (Collins, 2022; Guan & Goettker, 2024; Maljkovic & Nakayama, 1994; Manassi et al., 2019; Yashar et al., 2013), motion (Czoschke et al., 2019; Fischer et al., 2020), face (Hsu & Lee, 2016; Kondo et al., 2022; Liberman et al., 2014; Liberman et al., 2018; Turbett et al., 2022; Van der Burg et al., 2021), and attractiveness evaluation (Ho & Newell, 2020; Kim et al., 2019; Pegors et al., 2015; Xia et al., 2016) tasks, as well as in gaze direction (Alais et al., 2018; Bliss et al., 2023; Goettker & Stewart, 2024). Some studies refer to sequential effects, whereas other refer to serial dependence, because the former term encompasses the latter, we use ‘sequential effects’ from here on.

Visual perception is also modulated by covert attention. To process rich and complex visual environments effectively, covert attention allocates limited bioenergetic resources according to task demands in the absence of gaze shifts (Carrasco, 2011; Lennie, 2003; Pestilli & Carrasco, 2005). Endogenous attention is voluntary, goal-driven, flexible, and sustained, whereas exogenous attention is involuntary, stimulus-driven, automatic, and transient. Both improve performance (reviews, Carrasco, 2011; Carrasco & Barbot, 2014) and alter appearance (review, Carrasco & Barbot, 2019) across a wide range of visual tasks. Endogenous attention is more flexible than exogenous attention, modulating perceptual sensitivity adaptively as a function of task demands. For example, it enhances contrast sensitivity (Jigo & Carrasco, 2020) and increases the gain (Fernández et al., 2022) across a wider range of spatial frequencies than exogenous attention. Furthermore, endogenous attention improves performance in texture segregation tasks regardless of spatial resolution (Barbot & Carrasco, 2017; Yeshurun et al., 2008), whereas exogenous attention can improve or impair performance depending on spatial resolution (Carrasco et al., 2006; Yeshurun & Carrasco, 1998). It also facilitates temporal-order judgments, whereas exogenous attention can impair them (Hein et al., 2006). Finally, endogenous attention scales with cue validity to allocate resources accordingly, but exogenous attention does not (Giordano et al., 2009). Thus, we investigated whether and how the flexibility of endogenous attention extends to trial-by-trial adjustments of sequential effects and varies across the visual field.

Visual performance is not homogeneous around the visual field; in human adults, it is better at the horizontal than the vertical meridian (horizontal-vertical anisotropy, HVA) and better at the lower than the upper vertical meridian (vertical meridian asymmetry, VMA). These performance fields are present in many tasks, including contrast sensitivity (Abrams et al., 2012; Baldwin et al., 2012; Cameron et al., 2002; Carrasco et al., 2001; Corbett & Carrasco, 2011; Himmelberg et al., 2020; Kwak, Lu, & Carrasco, 2024; Lee & Carrasco, 2025), visual acuity (Barbot et al., 2021; Kwak et al., 2023), spatial resolution (Altpeter et al., 2000; Carrasco et al., 2002; Greenwood et al., 2017), and motion (Fuller & Carrasco, 2009; Tünçok, Kiorpes, & Carrasco, 2025). They also emerge in mid-level processes such as texture segregation (Talgar & Carrasco, 2002; Wang et al., 2020), size (Schwarzkopf, 2019), and crowding (Greenwood et al., 2017; Kurzawski et al., 2023; Petrov & Meleshkevich, 2011), as well as high-level tasks such as letter recognition (Mackeben, 1999), numerosity perception (Chakravarthi et al., 2022), face perception (Afraz et al., 2010; Kim & Chong, 2024), word identification (Tsai et al., 2024), and visual short-term memory (Montaser-Kouhsari & Carrasco, 2009).

Performance fields are not easily reshaped; despite its flexibility, endogenous attention benefits performance at all locations to a similar degree and does not compensate for these asymmetries (Lee & Carrasco, 2026; Purokayastha et al., 2021; Tünçok, Carrasco, & Winawer, 2025). Performance fields are also resistant to exogenous attention (Cameron et al., 2002; Carrasco et al., 2001; Roberts et al., 2016, 2018) and endogenous temporal attention (Fernández et al., 2019). Moreover, presaccadic attention, which enhances processing at the impending saccade target, can even exacerbate them by enhancing contrast sensitivity most at the horizontal meridian and least at the upper vertical meridian (Hanning et al., 2022, 2024; Kwak et al., 2025; Kwak, Zhao, et al., 2024).

The relation between covert spatial attention and sequential effects remains unknown. Some have reported that sequential effects decrease when no attention is allocated to previously experienced stimuli (Fischer & Whitney, 2014; Fritsche & de Lange, 2019; Rafiei et al., 2021), but others have reported an absence of such dependence (Fornaciai & Park, 2018; Goettker & Stewart, 2022). However, these studies did not monitor eye movements, or cleanly isolate endogenous from exogenous attention (Carrasco, 2011; Carrasco & Barbot, 2014; Chica et al., 2013; Olivers, 2025), rendering their findings inconclusive. Few studies have explored sequential effects with properly operationalized attention. They have shown similar sequential effects in performance, but differential oculomotor responses. For example, studies on presaccadic attention showed that performance in orientation discrimination tasks remains stable regardless of whether participants were performing spontaneous or cue-driven saccades on the previous trial (Tyralla & Zimmermann, 2025). However, performance improves when the saccade in the previous trial was directed to the same location (White et al., 2013). Likewise, when deploying covert attention to a specific time point, the effects of temporal attention on performance are similar across trials regardless of temporal attention and expectation on the previous trial (Duyar & Carrasco, 2025b). However, microsaccades are biased by expectations formed in the previous trial (Duyar & Carrasco, 2025a).

Sequential effects may be stronger at peripheral than central locations (Kandemir & Olivers, 2026; but see Suárez-Pinilla et al., 2018), given that perceptual sensitivity decreases with eccentricity (reviews, Himmelberg, Winawer, & Carrasco, 2023; Olivers, 2025; Strasburger et al., 2011), this peripheral increase would suggest a heavier reliance on prior experience when perceptual sensitivity decreases. Whether this explanation generalizes to polar angle differences remains unknown, as different mechanisms underlie sensitivity across eccentricity and around polar angle (Jigo et al., 2023; Xue et al., 2024, 2026).

**Figure 1.**
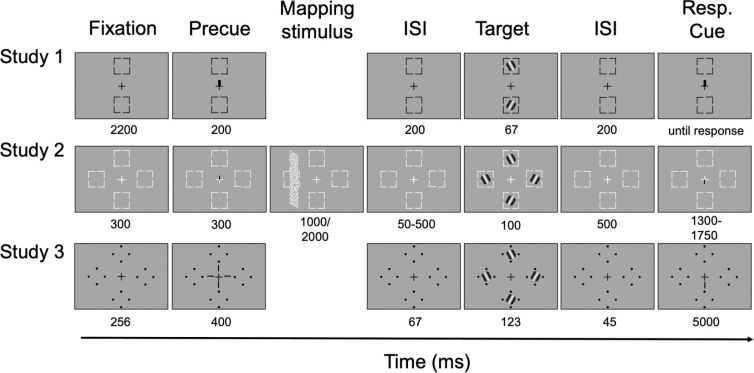
Trial sequence: Panels illustrate example trials for valid (Study 1, top panel), invalid (Study 2, middle panel), and neutral (Study 3, bottom panel) cue conditions, determined by the relation between the precue and the response cue location. The number below each frame indicates the duration (in ms). Stimulus size, spatial frequency, and background luminance are not to scale.

To investigate whether and how sequential effects influence endogenous attentional modulation around polar angle, we assessed sequential effects in three published studies of orientation discrimination in (1) response repetition and (2) perceptual sensitivity (Figure 1). Although stimulus parameters varied across these studies (e.g., stimulus size, eccentricity, number of possible target locations; see Methods for details), we evaluated sequential effects along three key dimensions (Manassi et al., 2023; Manassi & Whitney, 2024): (1) spatial, as the distance between successive targets increases, sequential effects decrease (Collins, 2019; Fischer & Whitney, 2014; Fornaciai & Park, 2018; Luo et al., 2022); featural, as the similarity between consecutive targets decreases, sequential effects decrease (Barbosa & Compte, 2020; Fischer & Whitney, 2014; Liberman et al., 2014; Turbett et al., 2021); (3) attentional, with stronger sequential effects produced by valid than invalid attention conditions in the previous target (Fischer & Whitney, 2014; Fritsche & de Lange, 2019; Kim et al., 2020; Rafiei et al., 2021). Furthermore, we also examined whether sequential effects depend on the correctness of the previous (*n* −1) trial (Danielmeier & Ullsperger, 2011; Little & Clifford, 2025). Given the flexible nature of endogenous attention, participants may adapt strategies based on prior performance. Namely, they may deploy more resources to the target when the previous trial was incorrect than when it was correct. Additionally, this compensatory mechanism may enhance performance more at locations with poorer sensitivity (i.e., the upper vertical meridian).

The aims of this study were twofold: First, we assessed the typical sequential effects in “response repetition (bias)”, in which the response to the current trial is biased towards the previous (*n* −1) one. Second, and more importantly, we examined if sequential effects would modulate the performance across locations in endogenous attention tasks.

## EXPERIMENTS

### Materials and Methods

This study reanalyzed data from three published studies. All participants had normal or corrected-to-normal vision. The Institutional Review Board at New York University approved the experimental procedures, and all participants provided informed consent before participating. We analyzed data from twelve adults (mean age: 28), including author HHL, who participated in Study 1 (Lee & Carrasco, 2026), nine adults (mean age: 28.5) who participated in Study 2 (Tünçok, Carrasco, & Winawer, 2025) and eighteen of the 20 participants (mean age: 27.3, data from the remaining two participants could not be recovered) in Experiment 2 of Study 3 (Purokayastha et al., 2021; data from the remaining two participants could not be recovered).

### Stimuli and Apparatus

Stimuli in all experiments were generated using MATLAB (MathWorks, Natick, MA) and Psychophysics Toolbox (Brainard, 1997; Pelli, 1997), and eye movements were monitored using an EyeLink 1000 (SR Research, Ottawa, ON, Canada) at a sampling rate of 1000 Hz to ensure fixation. Other details are specified below as well as in the original papers.

#### Study 1

The target Gabor (4° diameter, 5 cycles per degree (cpd), 1.25° full width at half maximum) was presented at four cardinal locations (left, right, upper, and lower), each positioned 8° from fixation (center-to-center). Four black placeholders (0.16° length × 0.06° width) were located 0.5° from the edge of the Gabor. A central fixation cross, consisting of a plus sign (0.25° length × 0.06° width), remained on the screen throughout the trial. The endogenous attentional cue (0.75° length × 0.2° width) was presented at fixation. Each participant completed two sessions.

Participants were seated in a dimly lit, sound-attenuated room, with their head stabilized on a chinrest at a viewing distance of 57 cm. Stimuli were displayed on a gamma-corrected 20-inch ViewSonic G220fb CRT monitor (ViewSonic Corporation, Brea, CA) with a resolution of 1280 × 960 pixels and a refresh rate of 100 Hz.

#### Study 2

The target Gabor (3° diameter, 4 cpd, 1° full width at half maximum) was presented at four cardinal locations (left, right, upper, and lower), each positioned 6° from fixation (center-to-center). Four white placeholders (0.22° length × 0.05° width) were located 0.11° from the edge of the Gabor. A white central fixation cross, consisting of a plus sign (0.35° length × 0.02° width), remained on the screen throughout the trial. The endogenous attentional cue was black and occupied one of the four arms of the fixation.

Participants lay supine in a 3T Siemens MAGNETOM Prisma MRI scanner (Siemens Medical Solutions, Erlangen, Germany); their heads were secured with pads to reduce head motion. Participants viewed the screen through an angled mirror mounted on the head coil. The total viewing distance (eye to mirror plus mirror to screen) was 86.5 cm. Stimuli were displayed on a ProPixx DLP LED projector with a linear gamma table (VPixx Technologies Inc., Saint-Bruno-de- Montarville, QC, Canada). The projected screen (60 × 36.2 cm) had a resolution of 1920 × 1080 with a refresh rate of 60 Hz.

#### Study 3

The target Gabor (3.2° diameter, 4 cpd, ~1° full width at half maximum) was presented at four cardinal locations (left, right, upper, and lower), each positioned 6.4° from fixation (center-to-center). Four placeholders, each composed of four black dots (0.05° radius), were arranged in a circle 0.5° from the edge of an upcoming Gabor patch stimulus. A central fixation cross, consisting of a plus sign (0.5° length), remained on the screen throughout the trial. The endogenous attentional cue was either a single 0.88° line or four 0.28° lines (all 0.14° thick), which were 0.38° from the center of the fixation cross to indicate the possible target locations.

Participants were seated in a dimly lit, sound-attenuated room, with their head stabilized on a chinrest at a viewing distance of 57 cm. Stimuli were displayed on a gamma-corrected Apple iMac MC413LL/A 21.5-in. desktop monitor (3.06 GHz Intel Core 2 Duo) with a resolution of 1280 × 960 pixels and a refresh rate of 90 Hz.

### Psychophysics task

All three tasks followed the same endogenous attention protocol but with different timing parameters (as specified in Figure 1). To equate discriminability across locations, before the endogenous attention task, participants’ performance was titrated for contrast thresholds in Study 1 and Study 3, and tilt angles in Study 2 for each location in a neutral-cue condition. More details can be found in the original studies. Feedback regarding accuracy was provided after each trial.

For the endogenous attention task in Studies 1 and 2, 20% of the trials had a neutral cue, in which the cue pointed to all possible target locations; the remaining 80% of the trials contained an informative attentional cue pointing toward a specific location (75% valid, 25% invalid). In Study 3, 50% of the trials were valid-cue trials, and the other 50% were neutral-cue trials.

### Statistical Analysis

We quantified the probability of a participant giving the same response based on whether the previous (*n* −1) trial was congruent (same) or incongruent (different) with the current trial. Task performance was indexed by *d′* [z(hit rate) – z(false alarm rate)] across conditions. Correct discrimination of clockwise trials was considered “hits” and incorrect discrimination of counter-clockwise trials was considered “false alarms”, consistent with previous studies (e.g., Jigo & Carrasco, 2020; Lee et al., 2024; Zhang et al., 2019). Bayes factors were estimated using the *BayesFactor* package in R (Morey et al., 2015) for 3-way interactions to evaluate evidence for or against an interaction among attention, polar angle location, and the sequential factor. Default Jeffreys–Zellner–Siow (JZS) priors were used, with fixed effects assigned a medium scale (*r*=0.5) and random effects a nuisance scale (*r*=1). *BF*_*10*_ values smaller than 0.33, 0.1, and 0.01 indicate moderate, strong, and very strong evidence favoring the null hypothesis, respectively (Raftery, 1999).

## Results

### Sequential effects on response repetitions

#### Study 1

In this experiment (Lee & Carrasco, 2026), target locations were blocked along either the vertical or the horizontal meridian; thus, each trial had two possible target locations. Participants also performed blocks following adaptation on different days, but the adaptation sessions were not analyzed in the current study.

**Figure 2.**
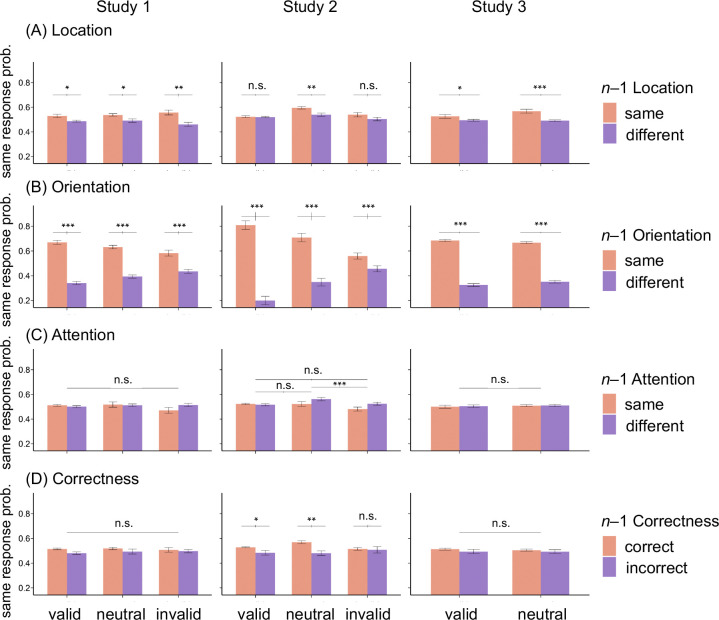
Probability of giving the same response in the orientation discrimination task when the (A) location, (B) target’s orientation, or (C) attentional cueing condition in the previous (*n* −1) trial was the same or different from the current trial. (D) probability of giving the same response in the task when performance of the previous (*n* −1) trial was correct or incorrect. Error bars within the bar plots depict ±1 SEM of the condition; error bars above the bars indicate ±1 SEM of the difference between conditions.

We excluded the first trial of each block and labeled the remaining trials based on whether the target location was the same as or different from that on the previous (*n* −1) trial. We conducted a two-way repeated-measures analysis of variance (rmANOVA) on the factors of attention condition (valid, neutral, invalid) and location repetition (same, different) while pooling all target locations in the analysis. For the location analysis (Figure 2A, **left panel**), there was a main effect of location repetition [*F*(1,11)=16.88, *p*=.002], no main effect of attention [*p*>.1], and a two-way interaction [*F*(2,22)=3.87, *p*=.036]. Paired *t*-tests revealed that participants gave more “same” responses in valid [*t*(11)=2.24, *p*=.047], neutral [*t*(11)=2.59, *p*=.025], and invalid trials [*t*(11)=4.39, *p*=.001] when the previous trial was at the same location than at the different location from the current one. The interaction was driven by the larger probability difference between the same and different conditions in the valid than the invalid condition [*t*(11)=2.31, *p*=.042].

For the feature analysis (Figure 2B, **left panel**), we conducted a two-way rmANOVA on the factors of attention (valid, neutral, invalid) and orientation repetition (same, different). There was a main effect of orientation repetition [*F*(1,11)=104.4, *p*<.001], no main effect of attention [*p*>.1] and a two-way interaction [*F*(2,22)=31.47, *p*<.001]. Paired *t*-tests showed that participants gave more “same” responses in valid [*t*(11)=11.58, *p*<.001], neutral [*t*(11,)=10.68, *p*<.001], and invalid trials [*t*(11)=5.05, *p*<.001] when the previous trial was at the same orientation than the different orientation from the current one. Here, a larger probability difference between the same and different orientation trials was observed in the valid than neutral condition [*t*(11)=5.36, *p*<.001], which in turn was larger in the neutral than invalid condition [*t*(11)=3.43, *p*=.006].

For the attention analysis (Figure 2C, **left panel**), a two-way rmANOVA on the factors of attention (valid, neutral, invalid) and attention repetition (same, different) yielded neither main effects nor interactions [*ps*>.1]. Lastly, for the correctness analysis (Figure 2D, **left panel**), a two-way rmANOVA on the factors of attention (valid, neutral, invalid) and *n* −1 correctness (correct, incorrect) revealed neither main effects nor interactions [*ps*>.05].

#### Study 2

Study 2 was a psychophysics experiment conducted in a functional magnetic resonance imaging (fMRI) scanner (Tünçok, Carrasco, & Winawer, 2025) featuring four possible target locations. We implemented the same labeling procedure and statistical analyses as in Study 1.

We conducted a two-way repeated-measures analysis of variance (rmANOVA) on the factors of attention condition (valid, neutral, invalid) and location repetition (same, different) (Figure 2A, **middle panel)**. There were main effects of location repetition [*F*(1,8)=10.56, *p*=.012], and attention [*F*(2,16)=7.94, *p*=.004] as well as a two-way interaction [*F*(2,16)=3.94, *p*=.041]. Participants gave more “same” responses in neutral [*t*(8)=4.49, *p*=.002] trials, but not in valid or invalid trials [*ps*>.1] when the previous trial was at the same location than at a different location from the current one. Indeed, the same-different location repetition had a larger probability difference in the neutral than the valid condition [*t*(11)=5.02, *p*=.001], with no other significant differences [*ps*>.1].

For the feature analysis (Figure 2B, **middle panel**), we conducted a two-way rmANOVA on the factors of attention (valid, neutral, invalid) and orientation repetition (same, different). There was a main effect of orientation repetition [*F*(1,8)=42.13, *p*<.001], no main effect of attention [*p*>.1] and a two-way interaction [*F*(2,16)=88.14, *p*<.001]. Participants gave more “same” responses in valid [*t*(8)=8.91, *p*<.001], neutral [*t*(8)=5.7, *p*<.001], and invalid trials [*t*(8)=2.37, *p*=.045] when the previous trial shared the same orientation than a different location from the current one. As in Study 1, a larger probability difference between the same and different orientation trials was observed in the valid than neutral condition [*t*(8)=7.2, *p*<.001], which in turn was larger than in the invalid condition [*t*(8)=7.92, *p*=.006].

For the attention analysis (Figure 2C, **middle panel**), we conducted a two-way rmANOVA on the factors of attention (valid, neutral, invalid) and attention repetition (same, different). There was a main effect of attention [*F*(2,16)=5, *p*=.021], but neither a main effect of attention repetition nor an interaction [*ps*>.05]. Post-hoc analysis revealed that participants gave more “same” responses in neutral than invalid trials [*t*(8)=5.81, *p*<.001], no other differences were observed [*ps*>.1].

Lastly, for the correctness analysis (Figure 2D, **middle panel**), a two-way rmANOVA on the factors of attention (valid, neutral, invalid) and *n* −1 correctness (correct, incorrect) yielded a main effect of correctness [*F*(1,8)=27.42, *p*<.001], no main effect of attention [*p*>.1] and only a marginal interaction [*F*(2,16)=3.52, *p*=.054].

#### Study 3

Study 3 (Purokayastha et al., 2021) included only valid and neutral conditions, omitting the invalid condition. Nonetheless, we conducted the same analysis procedure as in Studies 1 and 2: a two-way repeated-measures analysis of variance (rmANOVA) on the factors of attention condition (valid, neutral) and location repetition (same, different) (Figure 2A, **right panel**). There was a main effect of location repetition [*F*(1,17)=30.07, *p*<.001], no effect of attention [*p*>.1] and a two-way interaction [*F*(1,17)=8.12, *p*=.011]. Participants gave more same response in valid [*t*(17)=2.71, *p*=.015] and neutral [*t*(17)=5.6, *p*<.001] trials when the previous trial was at the same location rather than at a different location, which led to a larger probability difference between the same and different condition in the valid than the neutral condition [*t*(17)=2.85, *p*=.011].

For the feature analysis (Figure 2B, **right panel**), we conducted a two-way rmANOVA on the factors of attention (valid, neutral) and orientation repetition (same, different). There was a main effect of orientation repetition [*F*(1,17)=1182, *p*<.001], no main effect of attention [*p*>.1] and a two-way interaction [*F*(1,17)=10, *p*=.006]. Participants gave more “same” responses in valid [*t*(17)=26.47, *p*<.001] and neutral [*t*(17)=31.39, *p*<.001] trials when the previous trial shared the same orientation as the current one, which led to a larger probability difference between the same and different condition in the valid than neutral condition [*t*(17)=3.16, *p*=.006]–the same pattern observed in Studies 1 and 2, except that there was no invalid condition in this study.

For the attention analysis (Figure 2C, **right panel**), a two-way rmANOVA on attention (valid, neutral) and attention repetition (same, different) yielded neither main effects nor an interaction [*ps*>.1]. Lastly, for the correctness analysis (Figure 2D, **right panel**), a two-way rmANOVA on attention (valid, neutral) and *n* −1 correctness (correct, incorrect) revealed neither main effects nor an interaction [*ps*>.1].

### Combined analysis for response repetition

To examine the consistency of our findings across studies and enhance statistical power, we combined the data from these three studies (for valid and neutral trials only, because Study 3 did not contain invalid trials) and conducted four analyses. We treated the probability of giving a “same” response as the dependent variable and conducted a series of 2-way rmANOVAs on the factors of attention (valid, neutral) and trial history–the location, orientation, attention repetition (same, different), and *n* −1 correctness (correct, incorrect).

**Figure 3.**
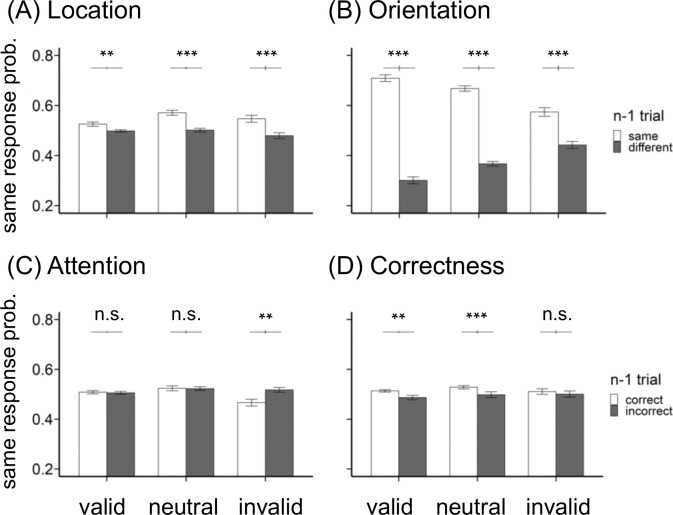
Probability of giving the same response in the orientation discrimination task when the (A) location, (B) target’s orientation, or (C) attentional cueing condition in the previous (*n* −1) trial was the same as or different from the current trial. (D) probability of giving the same response in the task when performance of the previous (*n* −1) trial was correct or incorrect. Error bars within the bars depict ±1 SEM of the condition; error bars above the bars indicate ±1 SEM of the difference between conditions. Valid and neutral conditions include data from Studies 1–3; the invalid condition includes only Studies 1 and 2, as Study 3 did not test invalid trials.

For the location repetition analysis (Figure 3A), results revealed main effects of location repetition [*F*(1,38)=44.28, *p*<.001] and attention [*F*(1,38)=10.17, *p*=.003], as well as an interaction [*F*(1,38)=17.38, *p*<.001]. Paired *t*-tests revealed that the probability difference of giving the “same” response when the previous location was the same vs. different was larger in the neutral than the valid condition [*t*(38)=4.16, *p*<.001]. Across Studies 1 and 2, participants gave more “same” responses when the previous location was the same than different in the invalid condition [*t*(20)=3.96, *p*<.001].

For the feature repetition analysis (Figure 3B), results revealed a main effect of orientation repetition [*F*(1,38)=310.8, *p*<.001], a marginal effect of attention [*F*(1,38)=3.83, *p*=.058], and an interaction [*F*(1,38)=37.5, *p*<.001]. Paired *t*-tests revealed that the probability difference of giving “same” responses when the previous orientation was the same vs. different was larger in the valid than the neutral condition [*t*(38)=6.12, *p*<.001]. Across Studies 1 and 2, participants also gave more “same” responses when the previous orientation was the same than different in the invalid condition [*t*(20)=5.34, *p*<.001].

For the attention repetition analysis (Figure 3C), results revealed a main effect of attention [*F*(1,38)=4.7, *p*=.036] but no other significant effects [*ps*>.1]. Participants gave more “same” responses in a current neutral than valid trials. Interestingly, in the invalid condition in Studies 1 and 2, participants gave more “same” responses when the previous trial was in a different attentional condition from the current one [*t*(20)=3.66, *p*=.002].

For the *n* −1 correctness analysis (Figure 3D), results revealed a main effect of correctness [*F*(1,38)=13.18, *p*<.001]; participants gave more “same” responses when the previous trial was correct than incorrect. No other effects were significant [*ps*>.1]. Interestingly, there was no difference in the probability of giving the “same” response in the invalid condition [*t*(20)=0.66, *p*=.514].

### Sequential effects on the performance

#### Study 1

We first analyzed whether participants gave “same” responses more often than “different” responses. By pooling all factors together, a paired *t*-test showed that participants gave a similar proportion of “same” and “different” responses [*t*(11)=1.24, *p*>.1], indicating that participants’ responses vary across trials and were not completely contingent on their response in the previous (*n* −1) trial.

**Figure 4.**
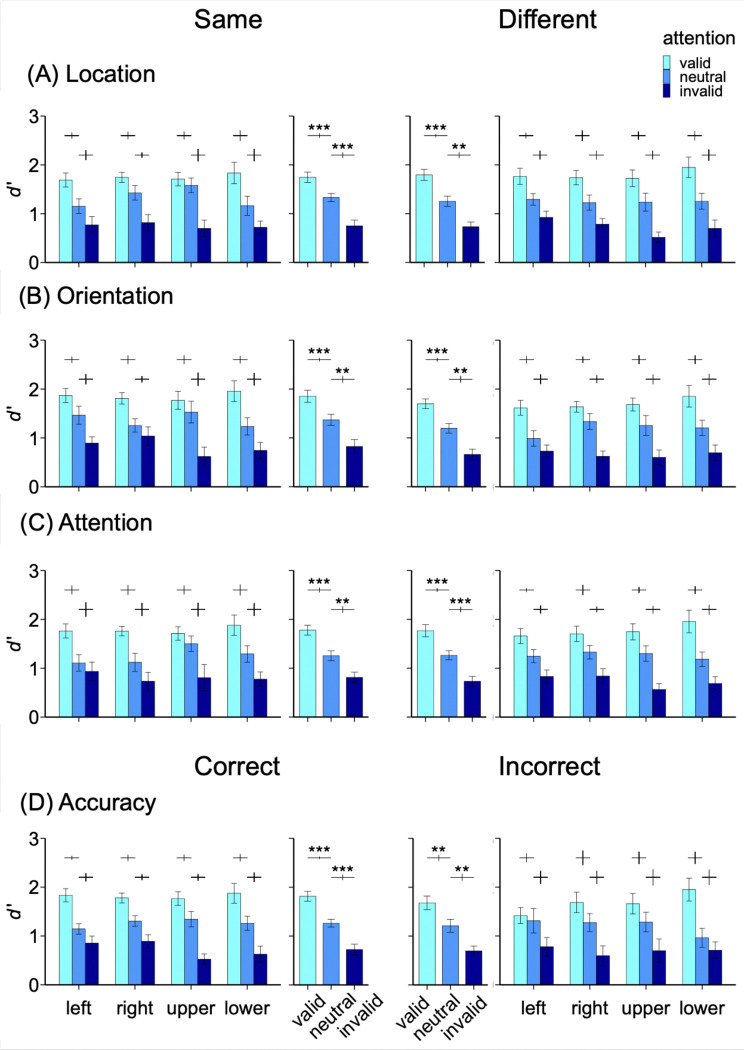
**Study 1.** Sensitivity in the orientation discrimination task when the (A) location, (B) target’s orientation, or (C) attentional cueing condition in the previous (*n* −1) trial was the same as or different from the current trial. (D) Sensitivity in the task when the performance of the previous (*n* −1) trial was correct or incorrect. For each plot, left and right represent the same and different (or correct and incorrect) conditions, respectively. The two middle columns indicate results averaged across locations. Error bars within the bars depict ±1 SEM of the condition; error bars above the bars indicate ±1 SEM of the difference between conditions.

To analyze whether target location repetition would enhance contrast perception (Figure 4A), we excluded the first trial of each block and labeled the remaining trials based on whether the target location was the same as or different from that on the previous (*n* −1) trial. We conducted a 3-way rmANOVA on the factors of attention condition (valid, neutral, invalid), location (left, right, upper, lower), and location repetition (same, different). Results revealed a main effect of attention [*F*(2,22)=51.91, *p*<.001], but neither main effects of location or location repetition [*ps*>.1] nor two-way [*ps*>.05] or three-way [*p*>.1, *BF*_*10*_=0.05] interactions.

For the feature analysis (Figure 4B), we conducted a 3-way rmANOVA on the factors of attention (valid, neutral, invalid), location (left, right, upper, lower), and orientation repetition (same, different). Results revealed a main effect of attention [*F*(2,22)=51.17, *p*<.001], but neither main effects of location or orientation repetition [*ps*>.05] nor two-way [*ps*>.1] or three-way [*p*>.1, *BF*_*10*_=0.13] interactions.

For the attention analysis (Figure 4C), we conducted a 3-way rmANOVA on the factors of attention (valid, neutral, invalid), location (left, right, upper, lower), and attention repetition (same, different). Results revealed a main effect of attention [*F*(2,22)=51.38, *p*<.001] but neither main effects of location or attention repetition [*p*>.1] nor two-way [*ps*>.1] or three-way [*p*>.1, *BF*_*10*_=0.09] interactions.

Lastly, for the correctness analysis (Figure 4D), we conducted a 3-way rmANOVA on the factors of attention (valid, neutral, invalid), location (left, right, upper, lower), and *n* −1 correctness (correct, incorrect). Results revealed a main effect of attention [*F*(2,22)=48.93, *p*<.001] but neither main effects of location or *n* −1 correctness [*p*>.1], nor two-way [*ps*>.1] or three-way [*p*>.1, *BF*_*10*_=0.23] interactions.

In addition, we analyzed the data separately by the first and second half of the blocks in each session and first versus the second sessions, we did not observe any interactions among attention, location, and the sequential effect (*ps*>.1), supporting the lack of interaction between trial history and attentional effects across locations. To summarize, an effect of attention was observed across all analyses: performance was higher in the valid condition and lower in the invalid condition than in the neutral condition. However, there were no significant effects of location or of sequential effects on target location.

#### Study 2

**Figure 5.**
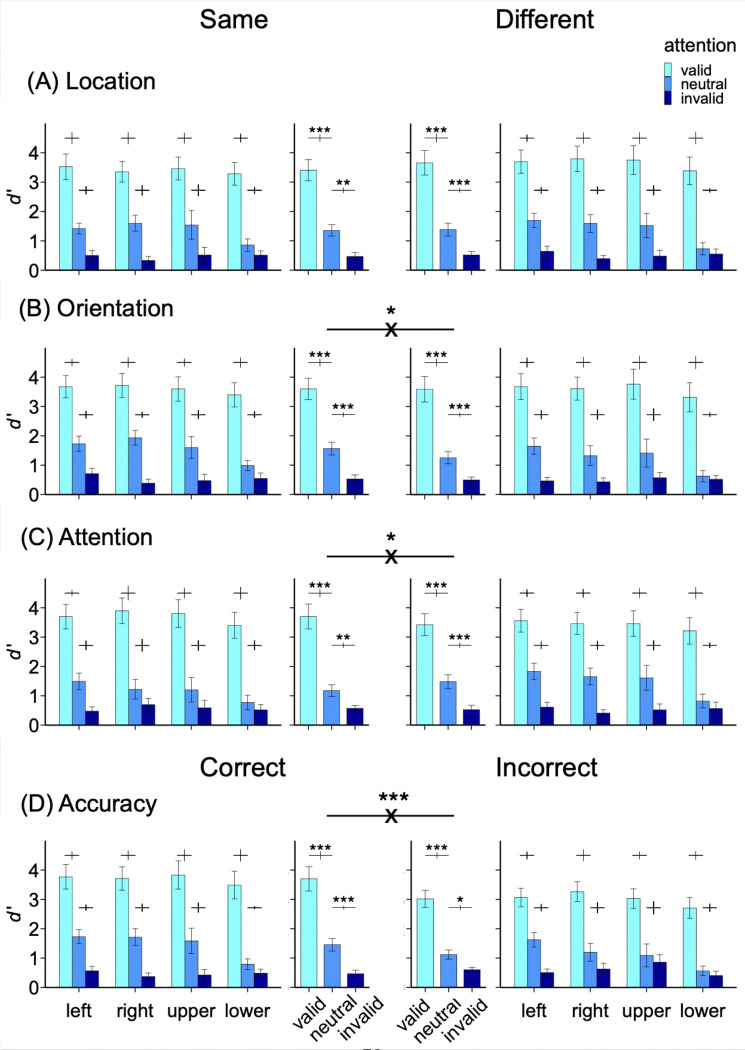
**Study 2.** Sensitivity in the orientation discrimination task when the (A) location, ((B) target’s orientation, or (C) attentional cueing condition in the previous (*n* −1) trial was the same as or different from the current trial. (D) Sensitivity when the performance of the previous (*n* −1) trial was correct or incorrect. For each panel, left and right bars represent the same and different (or correct and incorrect) conditions, respectively. The middle two columns indicate the results averaged across locations. Error bars within the bars depict ±1 SEM of the condition; error bars above the bars indicate ±1 SEM of the difference between conditions.

For the location analysis (Figure 5A), results revealed a main effect of attention [*F*(2,16)=72.69, *p*<.001], but neither main effects of location or location repetition [*ps*>.1], nor two-way [*ps*>.05] or three-way [*p*>.1, *BF*_*10*_=0.06] interactions.

For the feature analysis (Figure 5B), results revealed a main effect of attention [*F*(2,16)=72.92, *p*<.001], but not of location or orientation repetition [*ps*>.1]. There was a two-way interaction between attention and orientation repetition [*F*(2,16)=4.44, *p*=.029], where the neutral condition had a higher *d′* in the repeated orientation than the non-repeated orientation [*t*(8)=3.36, *p*=.01] but no difference was observed in the valid or invalid conditions [*ps*>.1]. There were no other two-way [*ps*>.05] or three-way [*p*>.1, *BF*_*10*_=0.06] interactions.

For the attention analysis (Figure 5C), results revealed a main effect of attention [*F*(2,16)=72.11, *p*<.001], but not of location or attention repetition [*ps*>.1]. There was a two-way interaction between attention and attention repetition [*F*(2,16)=5.32, *p*=.017], where the valid condition had a higher *d′* in the repeated attention than the non-repeated condition [*t*(8)=3.97, *p*=.004] but no difference emerged in the neutral or invalid conditions [*ps*>.1]. There were no other two-way [*ps*>.1] or three-way [*p*>.1, *BF*_*10*_=0.07] interactions.

For the correctness analysis (Figure 5D), results revealed main effects of attention [*F*(2,16)=77.93, *p*<.001] and *n* −1 correctness [*F*(1,8)=7.34, *p*=.027], but not of location [*p*>.05]. There was a two-way interaction between attention and *n* −1 correctness [*F*(2,16)=11.51, *p*<.001], where the valid condition had a higher *d′* following a correct trial than an incorrect trial [*t*(8)=4.43, *p*=.002], but no difference was found in the neutral or invalid conditions [*ps*>.05]. There were no other two-way [*ps*>.1] or three-way [*p*>.1, *BF*_*10*_=0.11] interactions.

To summarize, an effect of attention was observed across all analyses: performance was higher in the valid condition and lower in the invalid condition than the neutral condition. Moreover, sensitivity increased during the neutral condition when the previous target shared the same orientation, and during valid conditions when the preceding trial also had a valid cue. No significant interactions were observed between location and sequential effects.

#### Study 3

**Figure 6.**
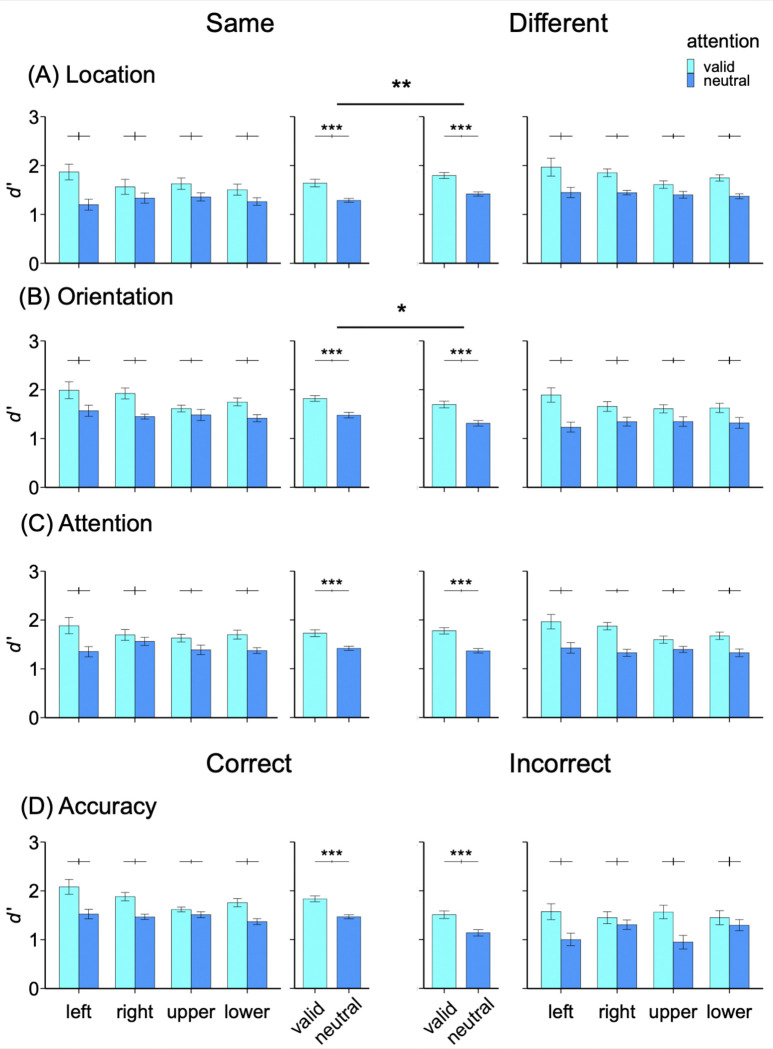
**Study 3.** Sensitivity in the orientation discrimination task when the (A) location, (B) target’s orientation, (C) attentional cueing condition in the previous (*n* −1) trial was the same as or different from the current trial. (D) Sensitivity when the performance of the previous (*n* −1) trial was correct or incorrect. For each panel, left and right bars represent the same and different (or correct and incorrect) conditions, respectively. The figures in the two middle columns indicate the results averaged across locations. Error bars within the bars depict ±1 SEM of the condition; error bars above the bars indicate ±1 SEM of the difference between conditions.

For the location analysis (Figure 6A), results revealed main effects of attention [*F*(1,17)=58.14, *p*<.001] and location repetition [*F*(1,17)=9.42, *p*=.007], where non-repeated trials had a higher *d′* than repeated ones. There was neither a main effect of location [*p*>.1] nor any two-way [*ps*>.05] or three-way [*p*>.1, *BF*_*10*_=0.12] interactions.

For the feature analysis (Figure 6B), results revealed main effects of attention [*F*(1,17)=79.43, *p*<.001] and orientation repetition [*F*(1,17)=6.39, *p*=.022], where repeated trials had a higher *d′* than non-repeated ones. There was neither a main effect of location [*p*>.1] nor any two-way [*ps*>.05] or three-way [*p*>.1, *BF*_*10*_=0.17] interactions.

For the attention analysis (Figure 6C), results revealed a main effect of attention [*F*(1,17)=81.45, *p*<.001], but neither main effects of location or attention repetition [*ps*>.1] nor any two-way [*ps*>.1]or three-way [*p*>.1, *BF*_*10*_=0.33] interactions.

For the correctness analysis (Figure 6D), results revealed main effects of attention [*F*(1,17)=59.65, *p*<.001] and of *n* −1 correctness [*F*(1,17)=29.28, *p*<.001], but not of location [*p*>.1]. There was a three-way interaction [*F*(3,51)=3.92, *p*=.014]. Paired *t*-tests showed that in the *n* −1 correct condition, the attentional benefit at the upper vertical meridian was smaller than at both the left [*t*(17)=3.37, *p*=.004] and right [*t*(17)=2.19, *p*=.043] horizontal meridians, but no other comparisons were significant [*ps*>.1]. In the *n* −1 incorrect condition, in contrast, the attentional benefit at the upper vertical meridian was greater than at both the right horizontal [*t*(17)=2.56, *p*=.02] and lower vertical meridians [*t*(17)=2.27, *p*=.037], but no other comparisons were significant [*ps*>.05].

In sum, there was an effect of attention across all analyses, whereby performance was higher in the valid condition, followed by the neutral and invalid conditions. In general, sensitivity (*d′*) was higher when the previous target appeared at a different location and when the previous trial shared the same orientation. Whereas no interactions between location and sequential effects emerged in the location, feature, or attention analyses, a significant interaction was found in the correctness analysis. Specifically, performance at the upper vertical meridian was lower than at other locations when the previous trial was correct, but higher when the previous trial was incorrect.

### Combined analysis for performance

#### The attention benefit

To examine the consistency of our findings across studies and enhance statistical power, we combined the data from the three studies and conducted four analyses. We treated the attentional benefit (valid *d′* – neutral *d′*) as the dependent variable and conducted a series of 2-way rmANOVAs on the factors of location (left, right, upper, lower), and trial history’s (*n* −1) location, orientation, attention (same, different), or correctness (correct, incorrect).

**Figure 7.**
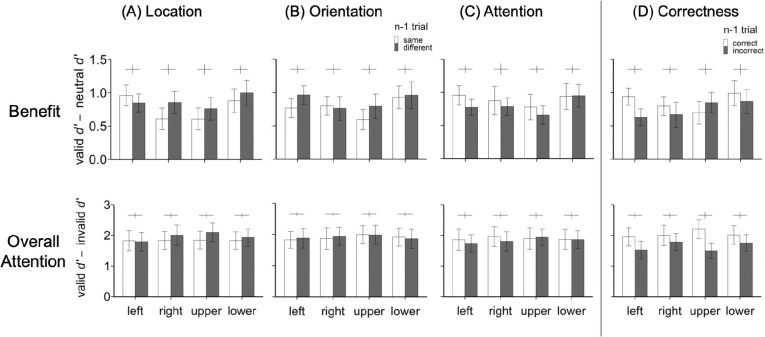
The benefit and overall attention effect (bottom panel) in the orientation discrimination task when the (A) location, (B) target’s orientation, or (C) attentional cueing condition in the previous (*n* −1) trial was the same as or different from the current trial. (D) The benefit and overall attention effect when the performance of the previous (*n* −1) trial was correct or incorrect. The top panels depict the attentional benefit and the bottom panels depict the overall attentional effect. Error bars within the bars depict ±1 SEM of the condition; error bars above the bars indicate ±1 SEM of the difference between the conditions.

For the location (Figure 7A, top panel), feature (Figure 7B, top panel), attention repetition (Figure 7C, top panel) and *n* −1 correctness analyses (Figure 7D, top panel), results revealed no main effects of location [*ps*>.05] or trial history factors [*ps*>.1] nor any two-way interactions [*ps*>.1, all *BF*_*10*_<0.33]. These results further show that the attentional benefit was stable and did not vary around the visual field, nor did it vary with the history of the prior trial.

#### The overall attention effect

To examine the consistency of attentional modulation, as reflected by the overall attention effect, we evaluated the validity effect (valid *d′* - invalid *d′*) across the two studies that contained invalid trials. We conducted a series of 2-way rmANOVAs on the factors of target location (left, right, upper, lower), and trial history’s (*n* −1) location, orientation, attention (same, different), or correctness (correct, incorrect).

For the location (Figure 7A, bottom panel), feature (Figure 7B, bottom panel), and attention repetition analyses (Figure 7C, bottom panel), results revealed no main effects of repetition or location [*ps*>.05] nor any two-way interactions [*p*>.1, all *BF*_*10*_<0.1]. These results further demonstrate that the overall attention effect was stable, regardless of the location, orientation, or attentional condition in the previous trial.

For the correctness analysis (Figure 7D, bottom panel), there was a main effect of *n* −1 correctness, where the overall attentional effect was higher if the previous trial was correct than if it was incorrect [*F*(1,20)=12.54, *p*=.002], but neither a main effect of location [*p*>.1] nor an interaction emerged [*p*>.1]. Thus, previous correct trials led to an overall larger attention effect than previous incorrect trials.

## Discussion

In all three studies analyzed here (Lee & Carrasco, 2026; Purokayastha et al., 2021; Tünçok, Carrasco, & Winawer, 2025), we observed a sequential effect in participants’ tendency to give a “same” response when the previous trial (*n* −1) shared the same location or orientation as the current trial (Figure 2), consistent with previous findings (e.g., Can & Collins, 2025; Murai & Whitney, 2021; St. John-Saaltink et al., 2016). Critically, endogenous attention significantly modulated performance to a similar degree around polar angle, and sequential effects did not influence the relation between attention and location. Thus, although the sequential effect biased the response toward the repeated target location and feature (Figures 2 and 3), it did not improve performance across locations in endogenous attention tasks (Figures 4–7).

For the response repetition analyses, participants were more likely to give a “same” response if the previous target was at the same location as the current one (Figures 2A and 3A). This aligns with the spatial dimension of sequential effects, in which stronger biases occur when consecutive targets overlap in space (Collins, 2019; Fischer & Whitney, 2014; Fornaciai & Park, 2018; Luo et al., 2022).

Additionally, a robust interaction emerged when the previous trial shared the same feature as the current one across three studies (Figures 2B and 3B) (Barbosa & Compte, 2020; Fischer & Whitney, 2014; Liberman et al., 2014; Turbett et al., 2021). Specifically, when the previous trial had the same orientation and the current trial was a valid condition, participants were more likely to give the same response than when orientations differed. Interestingly, this relation was modulated by attentional condition; for the first time, we observed that the response repetition probability difference between the same and different orientations declined as a function of cue validity (Figure 3B). This shows that participants adaptively tailored their strategy in response to task demands.

When the attentional condition was the same as the previous trial, it did not modulate the probability of giving the same response, except for the invalid condition when analyzing both studies together (Figure 3C). Note that our analysis evaluated whether the attentional cueing condition was repeated, rather than its validity *per se*. Thus, participants did not change their response strategy in valid and neutral conditions but did so in the invalid condition: if the current trial was invalid, participants were more likely to rely on their previous response if the previous trial was a different attentional condition.

As for trial correctness, more “same” responses were given if the previous trial was correct than incorrect (Figure 3D), a strategy participants adopted regardless of attentional condition. A correct response usually reinforces response repetition (Little & Clifford, 2025; St. John-Saaltink et al., 2016). However, this reinforcement did not benefit performance. Indeed, sequential effects on performance (sensitivity) were largely absent across all three studies, despite minor differences.

For the performance analyses, in Study 1, neither location nor its sequential history influenced performance. In Study 2, sequential effects emerged when either location or target orientation was repeated, yet these effects were uniform across polar angles. In Study 3, sensitivity increased when the previous target appeared at a different location or when the previous trial shared the same orientation; however, neither effect interacted with attention or location. Notably, performance at the upper vertical meridian was lower following correct trials but higher following incorrect trials. Additionally, a combined analysis across the three studies confirmed that the attention effects were invariant across polar angles and did not interact with sequential effects.

These results, supported by Bayes Factor analyses, provide the first evidence that endogenous covert spatial attention is invariant not only to location but also to sequential effects. Although endogenous attention is flexible (reviews, Carrasco, 2011; Carrasco & Barbot, 2014), here, it did not allocate more resources to locations where discriminability is intrinsically poor (i.e., the vertical meridian, especially the upper vertical meridian), even when considering previous trial conditions and response correctness. Thus, the finding that endogenous attention enhances contrast sensitivity similarly around polar angle is robust across eccentricities (from 6° to 8°), target orientations (from 2.5° to 20° tilt), spatial frequencies (from 4 to 5 cpd), sizes (from 3° to 4° visual angle), and number of possible target locations (from 2 to 4) in these three studies (Lee & Carrasco, 2026; Purokayastha et al., 2021; Tünçok, Carrasco, & Winawer, 2025).

The finding that polar angle asymmetries are not modified by sequential effects aligns with their resilience to several fundamental visual processes. These include endogenous (Lee & Carrasco, 2026; Purokayastha et al., 2021; Tünçok, Carrasco, & Winawer, 2025) and exogenous (Cameron et al., 2002; Carrasco et al., 2001; Roberts et al., 2016, 2018) spatial attention, endogenous temporal attention (Fernández et al., 2019) and presaccadic attention, the latter of which even exacerbates these asymmetries (Hanning et al., 2022, 2024; Kwak et al., 2025; Kwak, Zhao, et al., 2024). However, recently, two visual phenomena—preview and adaptation—have been shown to alleviate performance asymmetries and render perception more homogeneous. First, the preview benefit is inversely related to polar angle asymmetries; it is largest at the upper vertical meridian and smallest at the horizontal meridian, indicating that the preview benefit helps compensate for performance asymmetries by integrating information across saccades (Liu et al., 2024). Second, the adaptation effect is stronger along the horizontal meridian than the vertical meridian, mitigating the horizontal-vertical asymmetry (HVA) in contrast sensitivity (Lee & Carrasco, 2025).

The present findings of no sequential effects on performance are apparently inconsistent with the stronger sequential effect in response bias in the periphery than in foveal vision (Kandemir & Olivers, 2026). These authors propose that sequential effects scale inversely with perceptual sensitivity due to spatially-tuned continuity fields and spatial pooling in the visual cortex (Fischer & Whitney, 2014; Manassi et al., 2023; Manassi & Whitney, 2024). Accordingly, we would have expected stronger sequential effects at the upper vertical meridian where sensitivity is worst. Our data, however, show no such amplification in performance, suggesting eccentricity-dependent findings do not generalize to polar angle asymmetries, which appear governed by distinct system-level computations (Xue et al., 2024, 2026).

By combining data from three studies examining endogenous attentional effects across the visual field, we identified consistent patterns—such as a general absence of overall sequential effects on performance—despite parameter variations, such as the number of target locations, stimulus size, spatial frequency, eccentricity, inter-stimulus interval (ISI), and the inclusion of invalid trials. However, we also observed minor differences in the statistical outcomes across the individual protocols. In Study 2, higher performance occurred on valid trials following a correct trial than an incorrect one. When combining Studies 1 and 2, a correct preceding trial led to a larger overall attentional effect in the subsequent trial, regardless of target location (Figure 7D, bottom panel), a phenomenon likely driven by higher sensitivity on valid trials (Figures 4D & 5D), even though this effect did not reach statistical significance in Study 1. Thus, successful responses may reinforce the effective deployment of endogenous attention, enhancing its effect in the subsequent trial (Danielmeier & Ullsperger, 2011). In Study 3, we observed a sequential effect at the upper vertical meridian when analyzing performance following correct versus incorrect trials: a smaller attention benefit when the previous trial was correct, but a larger attention benefit when the previous trial was incorrect at (Figure 6D). The protocol in Study 3 differed from Studies 1 and 2 in that cue validity was 100% (rather than 75%). Accordingly, participants were more likely to rely on the cue (Giordano et al., 2009; Kinchla, 1980; Mangun & Hillyard, 1990; Sperling & Melchner, 1978; Vossel et al., 2006). We speculate that participants reweighed their attention resources nonuniformly following an error, with the upper vertical meridian receiving more resources. Future research could systematically manipulate these varying parameters to precisely determine the specific factors responsible for these minor differences.

Overall, we observed sequential effects on response repetition probability but did not observe sequential effects on performance regarding location, target orientation, attention repetition, or *n* −1 correctness. When examining sequential effects or serial dependence, most studies have reported how the response was biased toward the previous stimulus, via adjustment or reproduction tasks (e.g., Ceylan & Pascucci, 2023; Kondo et al., 2022; Manassi et al., 2023; Samaha et al., 2019). Whereas we confirm this response repetition bias using orientation discrimination tasks (e.g., Can & Collins, 2025; Murai & Whitney, 2021; St. John-Saaltink et al., 2016), perceptual ‘sensitivity’ or ‘performance’ has rarely been evaluated alongside it. Thus, sequential effects on performance and on response repetition should not be directly compared. The current study revealed that neither the magnitude of endogenous spatial attentional benefits nor overall sensitivity was modulated by prior trial history across spatial, feature, or attentional dimensions. These results are consistent with an endogenous temporal attention orientation discrimination task: perceptual sensitivity was independent of the temporal attentional condition in the previous trial (Duyar & Carrasco, 2025a).

In contrast to the lack of sequential effects on performance in our covert endogenous attention tasks, presaccadic attention exhibits sequential effects based on location and feature analysis in a motion discrimination task (White et al., 2013), and eye movement patterns are modulated by the previous trial in an orientation reproduction task (Tyralla & Zimmermann, 2025). Presaccadic attention differs from endogenous attention in its task structure (involving eye movements or not), how they shape sensory tuning (Li et al., 2016, 2019, 2021), and their neural underpinnings (Fernández et al., 2023; Hanning et al., 2023). Our findings provide further evidence that these two types of spatial attention yield different perceptual outcomes and sequential effects.

The neural underpinnings of sequential effects remain a subject of debate (Manassi & Whitney, 2024; Pascucci et al., 2023). Relevant to the current study are fMRI findings showing that in orientation discrimination tasks, signals in V1 are biased toward the orientation presented on the preceding trial (St. John-Saaltink et al., 2016), and TMS findings demonstrating that stimulating the lateral prefrontal cortex can effectively reduce serial dependence (Bliss et al., 2023). It is possible that early visual cortex receives top-down signals from the prefrontal area and contributes to serial dependence (Cicchini et al., 2021; Manassi & Whitney, 2024). For endogenous attention, another top-down mechanism modulates fMRI BOLD signals in the early visual cortex: higher-order, fronto-parietal attentional regions send feedback information to the visual cortex, with effects diminishing in earlier visual areas (Beck & Kastner, 2014; Chica et al., 2013; Dugué et al., 2020; Lauritzen et al., 2009; Pestilli et al., 2011; Puckett & DeYoe, 2015; Tünçok, Carrasco, & Winawer, 2025). Moreover, TMS studies have revealed that the human homolog of the right frontal eye fields (rFEF+) is functionally necessary for endogenous attention, but early visual cortex is not (Fernández et al., 2023).

In contrast, polar angle asymmetries have been linked to optical and retinal factors (Kupers et al., 2019, 2022) and to the functional architecture of the early visual cortex (Benson et al., 2021; Himmelberg et al., 2021, 2022, 2025; Himmelberg, Tünçok, et al., 2023). Although cortical surface area contributes substantially and correlates with contrast sensitivity and acuity (Benson et al., 2021; Himmelberg et al., 2022), it does not fully account for these asymmetries (Himmelberg, Winawer, & Carrasco, 2023; Jigo et al., 2023). Additional factors, such as variations in the gain of sensory tuning, also play a role (Xue & Carrasco, 2024; Xue et al., 2026).

Whether serial dependence operates at the perceptual level (Burr & Cicchini, 2014; Cicchini et al., 2017; Collins, 2020; St. John-Saaltink et al., 2016) or post-perceptual decision level (Alais et al., 2017; Bliss et al., 2017; Ceylan et al., 2021; Fritsche et al., 2017) remains debated. Although not our primary focus, our findings support a decision-level account given that trial history altered response repetition probability but performance (*d′*) remained invariant. This creates a dissociation between perception and response strategy during endogenous attention tasks.

In conclusion, by analyzing three datasets of endogenous attentional effects across four different aspects of trial history, we demonstrate typical sequential effects on response repetition bias. However, despite its flexible nature, endogenous attention does not preferentially enhance contrast sensitivity at any specific location, even when accounting for the location, feature, attention, and correctness of the preceding trial. These findings reveal that visual performance fields are not reshaped by covert spatial attention, regardless of recent trial history or top-down modulation, underscoring the robustness of performance asymmetries in human visual perception.
